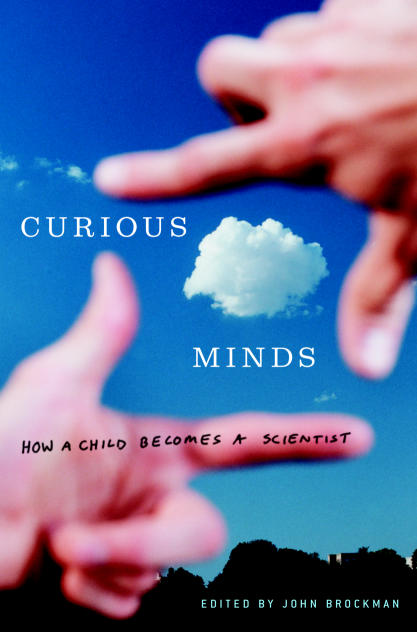# Turning to Science

**DOI:** 10.1371/journal.pbio.0030023

**Published:** 2005-01-18

**Authors:** Hemai Parthasarathy

## Abstract

A review of John Brockman's new book *Curious Minds: How a Child Becomes a Scientist.*

Do you want your child to grow up to be a scientist? If so, then by all means follow the roadmaps set down by the 27 scientists who contributed their personal histories to John Brockman's *Curious Minds: How a Child Becomes a Scientist*.

You could do worse than start with a family history rich in science, like Nicholas Humphrey whose grandfather, Nobel laureate A. V. Hill, remembers being startled by a solar eclipse while out rabbit hunting as a boy in England and having the presence of mind to smear the rabbit's blood over the glass from his pocket watch in order to watch the phenomenon in safety. Or, if you don't have a healthy lineup of scientific superstars in your family history, you could educate yourself about the wonders of science, like Murray Gell-Man's autodidactic father, and insist that your son at least try majoring in physics before settling on linguistics or archaeology. If you cannot be inspired to teach yourself quantum physics, you might also try the more commonplace achievement of a bad marriage to create an unstable emotional life for your daughter, so she might retreat into “solitary and bookish tendencies,” as the evolutionist Lynn Margulis did.

Perhaps you cannot bring yourself to actively steer your child into science, but hope this book might guide you to at least watch for the signs of future genius. You might watch for a young Freeman Dyson in his crib, bored by the stuffed animals and mobiles, occupying his time by adding infinite series of fractions. Or you might watch for your daughter sneaking into your study to read your medical reference books on the sly, as Janna Levin did before ultimately becoming a professor of physics. Then again, you could watch for your son to emulate David Buss by reaching high for a C+ grade point average in school, indulging in recreational drugs, and taking a night shift job at a truck stop, before a scientifically minded girlfriend and a fortuitous lottery-based scholarship to the University of Texas turned him to the pursuit of evolutionary psychology.

The pattern is clear: there is no pattern. And the book's strong contingent of psychologists is not shy about commenting on the dubious reliability of self-narrative. Steven Pinker writes, “Don't believe a word of what you read in this essay on the childhood influences that led me to become a scientist. Don't believe a word of what you read in the other essays, either….Recounting childhood influences is a mental process no less subject to quirks and errors than falling for the visual illusions on the back of a cereal box….None of us has taken part in the experiments that would isolate the causes of our choices in life.” Or as Nicholas Humphrey puts it more simply, “Each of us is who we are, and we must each have had *some* sort of childhood. Who's to say whether any particular factor carried the weight that our self-narrative now likes to attribute to it.”

This book, then, is not a guide to the prescientific childhood, but a set of memoirs bound by their common conclusion. Many of them are highly entertaining, some of them are self-conscious and pedantic. All of them highlight the passion of childish curiosity extending into adulthood, either by family tradition or because a mentor appeared at the right place and time to ensure that this curiosity was not abandoned. As the developmental psychologist Allison Gopnik notes, “I suspect that there are few reports of scientists with a childhood fascination for babies, because most of those children turned into nursery school teachers or children's librarians or just stay at home mothers….It seems to me now that I was destined to become either a psychologically minded philosopher or a philosophically minded pscyhologist. But given slightly different contingencies, I might have become a frustrated preschool teacher or faculty wife.”

We wish we could find prescriptions for guiding our children towards success, but the nature versus nurture debate has long since been replaced by the more sophisticated understanding of just how inextricably intertwined these two influences are in creating even a lowly zebrafish, let alone anything as behaviorally complex as a human being. This understanding can lead to frustration, as simple causes and cures become more elusive. Selling Mozart recordings for expectant mothers to play to their wombs can help assuage the fears and ignite the hopes of overachieving, yuppie parents, but there is little evidence that such interventions create the sought after intellectual advantage for the newborn.

Mostly what we see in these essays are stories about people—stories that people tell about themselves. Stories of childhoods interrupted by war, of mentors persecuted by McCarthyism. Would a book tracing the self-styled childhood influences of outstanding politicians or judges have been so different? Possibly fewer socially awkward children and less gadget-building in the mix, possibly not.

The contributors to this book are all outstanding, motivated scientific leaders who have chosen or stumbled into their intellectual paths. But I am not convinced that reading their brief reflections compares with the unique opportunity of interviewing Albert Einstein that John Brockman identified as one inspiration for this book—for turning what began as dinner party conversation into a methodical attempt at biographical anthology. Much of these pages are in fact dinner party conversation, whether it is Robert Sapolsky's horror of the white Southern gentlemen of Harvard's intellectual elite playing cards and drinking while dividing the spoils of sociobiology, or Allison Gopnik's personal relief that there were no prohibitions against sleeping with teaching assistants when she was a nubile college student. Some of it is heart-rendingly personal, as when Jaron Lanier talks about his futile attempt at age 11 to attract friends to his homemade electronic Halloween haunted house. All it really tells us is that there are as many ways of forming a scientist as there are of forming a human being.

**Figure:**